# Rapidly Growing Nodular Fasciitis in the Cheek of an Infant: Case Report of a Rare Presentation

**Published:** 2008-05-29

**Authors:** E. Hanson Lenyoun, June K. Wu, Bryan Ebert, Benn Lieberman

**Affiliations:** Columbia University College of Physicians and Surgeons, New York; Division of Plastic Surgery, Department of Surgery, Columbia University College of Physicians and Surgeons, New York; Department of Pathology, Columbia University College of Physicians and Surgeons, New York; Department of Oral Surgery, Columbia University College of Dental Medicine, New York

## Abstract

**Objective**: Nodular fasciitis is a benign proliferative spindle-cell lesion found in the subcutaneous fascia that presents as a rapidly growing mass with rich cellularity and mitotic activity, leading to it frequently being mistaken for a sarcoma. Histomorphologic features and immunohistochemical profile are useful in proper diagnosis. The treatment is complete excision, and recurrence is uncommon. A rare presentation and treatment of nodular fasciitis in the cheek of an infant is described here. **Methods**: The lesion was resected by direct transcutaneous approach, and the skin was closed with 2 layers of purse-string polydioxanone sutures followed by fast-absorbing gut sutures to reapproximate the epidermis. **Results**: Postoperatively, the patient had a wound dehiscence on the cheek, which healed secondarily with wound care. A small area of palpable tumor unable to be resected remained stable after 4 months of follow-up, and facial nerve function was preserved. **Conclusions**: Although infrequent in both children and the oral mucosa, nodular fasciitis should be considered in the differential diagnosis of facial tumors in infants and young children.

*Nodular fasciitis* is defined by the World Health Organization as a benign and reactive fibroblastic growth extending from the superficial fascia into the subcutaneous tissue or muscle.^1^ It is a tumor-like spindle-cell lesion that is often mistaken for a sarcoma as a result of its rapid growth, rich cellularity, and mitotic activity.^2^ The proliferating tissue is fundamentally composed of myofibroblasts, which are commonly found in granulation tissue, arterial intima, the stroma of desmoids, fibromatoses, angiofibromas, and soft-tissue sarcomas.^3^ Nodular fasciitis presents as a rapidly growing mass, occasionally with pain or tenderness. The treatment is complete excision and recurrence is rare. They are generally small, solitary, equally distributed between genders, and more common in the third to fifth decades of life.^4^ Lesions are commonly located on the extremities, occasionally on the trunk, and infrequently on the head and neck. Lesions in the orofacial region are rare but, when they do occur, are most commonly located in the skin of the face, parotid gland, buccal mucosa, labial mucosa, and tongue.^5^ We present an unusual presentation of a facial nodular fasciitis on an infant.

## REPORT OF A CASE

**Figure 1:**
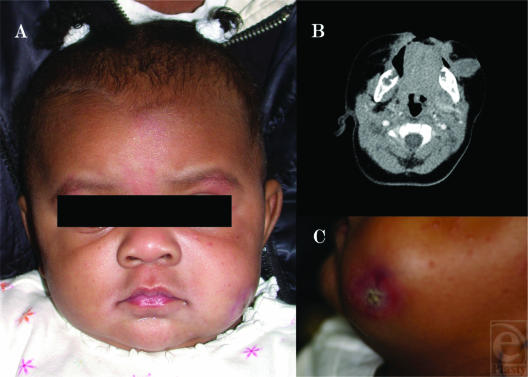
(A) The child with a rapidly growing tumor in her left cheek, shown as fullness when compared with the right cheek. (B) Computed tomographic scan showed the mass infiltrating the full thickness of the cheek. (C) Proliferating tumor with skin erosion in the overlying skin.

A 3-month-old girl with no significant medical history or history of trauma presented to an outside emergency department with a mass in the left cheek that had been rapidly growing over the course of 1 month (Fig 1A). On examination, the mass measured 2 × 3.5 cm, was firm and immobile, and extended the full thickness of the cheek from the skin to the oral mucosa. At this point, the skin was slightly distorted but no ulceration was noted. The oral mucosa remained intact. The presentation of this mass suggested malignancy, and the patient was referred to our institution for a multidisciplinary approach.

Over the course of several days, the patient was evaluated by pediatric oncology, pediatric surgery, pediatric plastic surgery, and oral and maxillofacial surgery. Computed axial tomographic scans were performed with contrast showing a solid mass in the subcutaneous tissues of the left cheek unable to be separated from the buccinator muscle (Fig 1B). The underlying mandible, neck, lungs, mediastinum, and bones all appeared normal. An intraoral incisional biopsy was performed under general anesthesia to rule out malignancy. Pathologic examination revealed cellular myofibroblastic proliferation consistent with nodular fasciitis.

**Figure 2:**
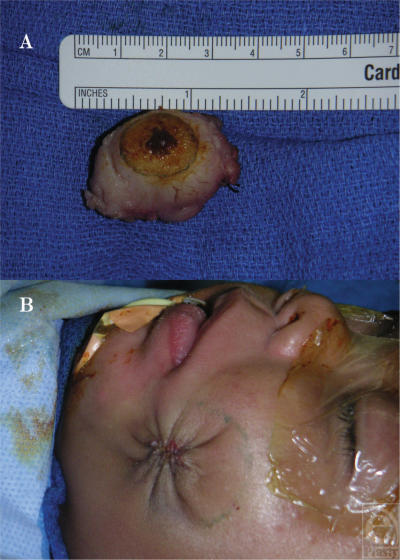
(A) Gross specimen following excision. (B) Immediate postoperative appearance of the incision with a purse-string closure.

The child was scheduled for resection of the lesion because of its continued proliferation and evidence of ulceration through the overlying skin (Fig 1C). The lesion was resected by direct transcutaneous approach through the ulcerated portion of the cheek. The tumor had developed deep to the buccinator muscle. It was dissected off the muscle fibers. A portion of mucosa measuring 1.6 × 1.5 cm was adherent to the lesion and was resected with the mass (Fig 2A). However, complete resection would not have been possible without the inclusion of an extensive area of mucosa, which would have left a complete through-and-through defect. The decision was made to leave the mucosa in place with some adherent residual tumor remaining as well. The small defect in the mucosa was closed with simple chromic sutures, the buccinator muscle fibers were reapproximated, and the skin was closed with 2 layers of purse-string PDS sutures, followed by fast-absorbing gut sutures to reapproximate the epidermis (Fig 2B).

**Figure 3:**
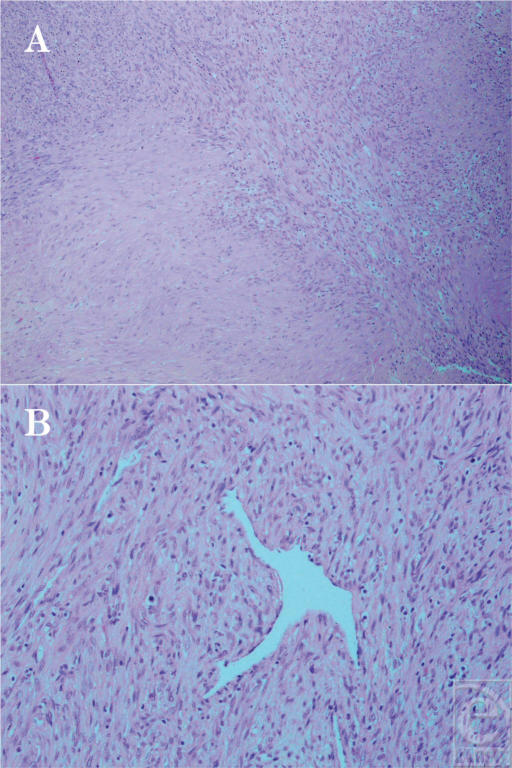
Histological examination ruled out a malignancy and showed myofibroblastic proliferation, consistent with nodular fasciitis (hematoxylin and eosin stain). (A) Low-power photomicrograph showing the zoned appearance of the tumor. In the lower left, myofibroblasts with a moderate amount of eosinophilic cytoplasm are seen arranged in a fascicular pattern. The upper right shows less well differentiated cells with larger nuclei and less cytoplasm (original magnification × 40). (B) Higher magnification photomicrograph from the less well differentiated area. A hemangiopericytoma-like appearance is seen, with arrangement of cells around thin-walled, irregularly branching blood vessels (original magnification × 100).

Surgical pathology confirmed the previous diagnosis of a benign myofibroblastic lesion. The histology showed a spindle cell lesion with a nodular growth pattern possessing eosinophilic cytoplasm and elongated tapered nuclei (Fig 3). There were scattered mitotic figures present. Immunohistochemical staining was positive for HHF-35, smooth muscle actin, and heavy caldesmon, and negative for cyto-keratin, S100, myoD1, and desmin, all of which were consistent with findings for nodular fasciitis reported in the literature.^6^ In another chromosomal study, karyotype revealed normal 46XX results with no abnormalities and FISH showed no rearrangements present.

**Figure 4:**
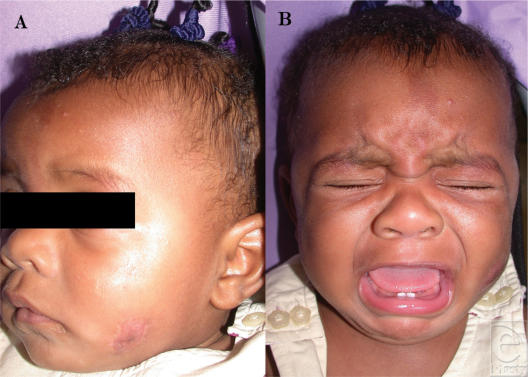
(A) Well-healed wound in the left cheek, 4-month postoperative. (B) Symmetrical facial expression demonstrating preservation of the facial nerve.

Postoperatively the patient had a wound dehiscence on the cheek, which healed secondarily with wound care. The small area of palpable tumor remained stable after 4 months of follow-up, and facial nerve function was preserved (Figs 4A and B).

## DISCUSSION

Konwaler et al.^7^ first described nodular fasciitis in 1955 under the name of subcutaneous pseudosarcomatous fibromatosis. It has also been called nodular fibrositis, subcutaneous fibromatosis, pseudosarcomatous fasciitis, and infiltrative fasciitis, as well as having been misclassified as several forms of sarcoma.^2^,^8^ Nodular fasciitis is a benign, idiopathic, reactive proliferation of myofibroblasts found in the subcutaneous fascia and presents as a rapidly growing mass, leading to it frequently being mistaken for a sarcoma. Nodular fasciitis is similar to sarcoma in that the lesions frequently extend along fascial planes and exhibit high cellularity, focal nuclear atypia, and mitoses. However, nodular fasciitis differs from sarcoma in several distinct manners and is not a presarcoma or transformative lesion.

Nodular fasciitis is extremely uncommon in children; it most commonly presents in individuals in their third to fifth decades of life. When seen in children, it may be confused with other more common soft tissue tumors such as fibroma, lipoma, desmoid tumor, sarcoma, chondroma, myxoma, malignant fibrous histiocytoma, schwannoma, atypical fibroxanthoma, or parotid tumor.^2^,^4^,^9^,^10^ Nodular fasciitis can be distinguished from sarcoma based on histology, molecular biology, and immunohistochemistry. Nodular fasciitis spindle cells contain vimentin, muscle-specific actin, and smooth-muscle–specific actin. They do not contain desmin, keritin, or S-100 protein, for which sarcoma spindle cells stain positive.^6^ Sarcoma spindle cell tumors may stain positive for smooth-muscle actin depending on their origin.^11^

Nodular fasciitis accounts for 0.025% of all pathologic diagnoses, with less than 4% of those cases occurring in children aged 0–9 years of age.^3^,^8^ This case report illustrates that when presented with a rapidly growing soft tissue tumor in a child, uncommon diagnoses need to be entertained as well. Tissue biopsy is useful to help guide diagnosis and treatment, even if it necessitates general anesthesia in a child. While benign, nodular fasciitis may still cause significant morbidities including ulceration, rapid growth, and functional impairment. Complete resection is the treatment of choice in cases of nodular fasciitis but is not always possible without the risk of serious morbidity. Partial excision is acceptable as residual lesion may resolve spontaneously and recurrence occurs rarely, if ever.^12^ In cases where proliferation is slow or absent, or where resection would be difficult or cause significant morbidity, surgery may be delayed in favor of close observation as lesions have been reported to resolve spontaneously or upon biopsy.^9^ However, growth or regression is unpredictable and prognosis upon resection is very good. Recurrence rate is quoted as 0.4% to 1% in the literature, but many believe that any recurrence indicates error in the initial diagnosis.^9^

This case demonstrates that, although infrequent in both children and the oral mucosa, nodular fasciitis should be considered in the differential diagnosis of facial tumors. Nodular fasciitis can be differentiated from other facial tumors, including sarcomatoid carcinoma, fibrosarcoma, leiomyosarcoma, and neurofibroma, by histopathological examination of the tissue. Accurate diagnosis and timely and comprehensive treatment and follow-up are necessary to avoid inappropriate measures and limit potential morbidities.
